# Outcomes of patients presenting with elevated tumor marker levels but negative gadoxetic acid-enhanced liver MRI after a complete response to hepatocellular carcinoma treatment

**DOI:** 10.1371/journal.pone.0262750

**Published:** 2022-01-27

**Authors:** Ka Eun Kim, Dong Hyun Sinn, Moon Seok Choi, Honsoul Kim

**Affiliations:** 1 Department of Radiology, Samsung Medical Center, Sungkyunkwan University School of Medicine, Seoul, Republic of Korea; 2 Department of Medicine, Samsung Medical Center, Sungkyunkwan University School of Medicine, Seoul, Republic of Korea; 3 Department of Health Science and Technology, SAIHST, Sungkyunkwan University, Seoul, Republic of Korea; Ospedale San Raffaele, ITALY

## Abstract

**Purpose:**

Hepatocellular carcinoma (HCC) patients usually achieve a complete response after treatment. This study was aimed to assess the clinical outcome of HCC patients who had achieved a complete response but later presented with elevated tumor marker levels without an identifiable recurrent tumor on gadoxetic acid-enhanced magnetic resonance imaging (MRI).

**Methods:**

We retrospectively reviewed the clinical outcome of 58 HCC treated patients who had achieved a complete response but later was referred to our institution’s multidisciplinary tumor board for a clinically suspected hidden HCC recurrence based on elevated tumor marker levels but negative gadoxetic acid-enhanced MRI. The imaging studies, tumor markers, and clinical information were reviewed. The total follow-up period was at least 15 months after the initial negative gadoxetic acid-enhanced MRI.

**Results:**

Follow-up imaging studies detected an HCC lesion in 89.7% (*n =* 52/58) of the patients within the study period, and approximately half of the tumors (46.2%, *n =* 24/52) developed within 3 months. The most frequent site of recurrence was the liver (86.5%; *n =* 45/52), but extra-hepatic metastasis was also common (19.2%; *n =* 10/52). In 5.8% (*n =* 3/52), HCC reoccurred in the combined form of intra-hepatic and extra-hepatic recurrence. Extra-hepatic metastasis alone occurred in 13.5% (*n =* 7/52) of patients.

**Conclusions:**

HCC frequently recurred within a short interval in patients who achieved a complete response to treatment in the presence of increased tumor marker levels, even if gadoxetic acid-enhanced MRI was negative. Under such circumstances, we suggest a short-term follow-up including, but not limited to, gadoxetic acid-enhanced MRI along with systemic evaluation.

## Introduction

Magnetic resonance imaging (MRI) plays an essential role in the detection and diagnosis of hepatocellular carcinoma (HCC) [1, 2]. MRI has excellent diagnostic performance for predicting complete pathological necrosis in HCC patients treated with loco-regional therapy [3]. Progress in HCC management has resulted in various treatment options, including surgical resection, liver transplantation, and local directive therapies, such as radiofrequency ablation, cryoablation, radioembolization, transarterial chemoembolization, and stereotactic body radiation therapy [4, 5]. Consequently, complete responses to treatment are more frequent in patients with HCC. The early detection of a recurrent tumor allows for possible reapplication of curative treatment modalities [6, 7]. Accordingly, the demand for effective post-treatment surveillance enabling early detection of potential tumor recurrence is also increasing.

Follow-up data after a complete response to treatment in HCC are very limited [6]. No widely accepted guidelines provide optimal follow-up protocols for HCC patients who achieve a complete response. In daily practice, follow-up is usually performed using tumor markers and imaging studies based on contrast-enhanced computed tomography (CT) and/or MRI [8–10], although the specific procedure and intervals are individualized by the physician [6, 11, 12].

Alpha-fetoprotein (AFP) and protein induced by vitamin K absence or antagonist-Ⅱ (PIVKA-Ⅱ) are well-established tumor markers used for HCC evaluation. They serve as an important tool for the early diagnosis of not only HCC but also tumor recurrence after treatment [13–15]. However, some patients present with elevated levels of these tumor markers, but HCC is not detectable by gadoxetic acid enhanced MRI. Gadoxetic acid is a widely used, sensitive imaging technique for early detection of HCC recurrence. Therefore, this situation might reflect the absence of a tumor, a non-detectable early tumor, or infiltrative HCC [16] and inevitably causes uncertainty because decisions on patient management are made with the possibility of a false-negative imaging study and/or false-positive tumor marker test. We considered the outcome of patients who had received treatment for HCC and achieved a complete response and a negative gadoxetic acid-enhanced MRI during follow-up but showed increased levels of tumor markers. This situation usually results in a clinical suspicion of a hidden recurrent tumor, but the clinical outcome and optimal management strategy in such patients remain unclear.

This study describes the clinical outcome of HCC patients who had achieved a complete response to treatment but later presented with elevated tumor marker levels without an identifiable recurrent tumor on gadoxetic acid-enhanced MRI.

## Materials and methods

### Study participants

This retrospective study was approved by the Institutional Review Board of Samsung Medical Center with a waiver of informed consent (IRB approval number: 2020-02-159). We reviewed patients who had been referred to our institution’s multidisciplinary tumor board [17] between September 2011 and October 2018 with a clinical suspicion of hidden HCC based on elevated tumor markers but negative gadoxetic acid-enhanced liver MRI. The inclusion criteria were as follows: (1) Patients with a medical history of treated HCC who achieved a complete response, (2) Patients referred to the multidisciplinary tumor board for clinically suspected hidden HCC recurrence based on documented elevation of serum AFP and/or PIVKA-Ⅱ levels. We did not define a specific threshold level of tumor markers but considered patients as candidates if the reason for referral was suspected hidden HCC due to unexplained tumor marker elevation. (3) Gadoxetic acid-enhanced MRI was performed within 4 weeks since blood testing of tumor markers, and the official MRI report concluded no evidence of viable HCC. (4) The patient’s case had been discussed by members of our multidisciplinary tumor board, and records of the proceedings stated that a radiologist specialized in HCC imaging had conducted a second review of the imaging studies, including MRI, and confirmed the results to be negative for viable HCC lesions. Complete response was determined based on modified RECIST guideline [18]. In case local treatment was performed and a remnant lesion existed then the absence of increase in size of the target lesion at the next follow up imaging study was also required for it to be determined to have achieved a complete response [19].

**Fig 1:**
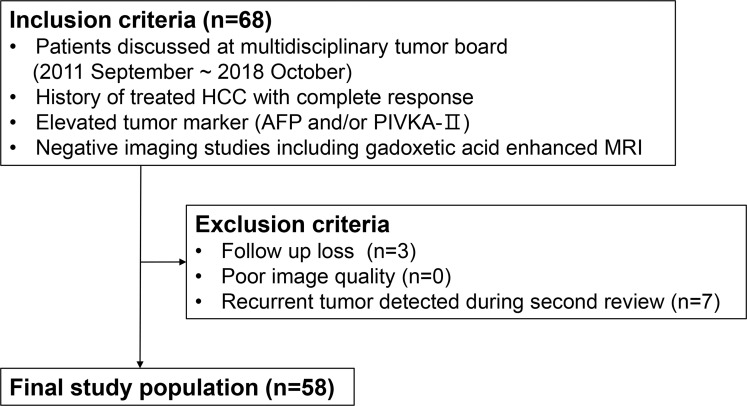
Flowchart showing the inclusion and exclusion criteria of the study.

The exclusion criteria were as follows: (1) a follow-up interval shorter than 15 months due to follow-up loss (*n =* 3); (2) poor-quality liver MRI not suitable for documentation of a negative imaging study (*n =* 0); or (3) a recurrent tumor detected by a radiologist during a second review by the multidisciplinary tumor board, indicating a false-negative interpretation of the MRI (*n =* 7) (Fig 1). This process defined a final study population of 58 patients (Table 1).

**Table 1:** Characteristics of the study population.

Parameter	Value
Sex/age	
Total (*n =* 58)	Mean±SD: 61.0±10.0, range: 27–84.
Male (*n =* 46)	Mean±SD: 61.0±9.4, range: 41–84.
Female (*n =* 12)	Mean±SD: 60.8±12.1, range: 27–76.
Underlying liver disease	
Hepatitis B	47 (81.0%, n = 47/58)
Hepatitis C	4 (6.9%, *n =* 4/58)
Alcohol abuse	3 (5.2%, *n =* 3/58)
Non-B or -C liver disease	4 (6.9%, *n =* 4/58)
Previous HCC staging	
Barcelona Clinic Liver Cancer staging 0	10 (17.2%, *n =* 10/58)
Barcelona Clinic Liver Cancer staging A	22 (37.9%, *n =* 22/58)
Barcelona Clinic Liver Cancer staging B	15 (25.9%, *n =* 15/58)
Barcelona Clinic Liver Cancer staging C	11 (19.0%, *n =* 11/58)
Previous HCC treatment	
Surgery only	7 (12.1%, *n =* 7/58)
Radiofrequency ablation only	3 (5.2%, *n =* 3/58)
Transarterial chemoembolization only	6 (10.3%, *n =* 6/58)
Radiation therapy only	1 (1.7%, *n =* 1/58)
Surgery + Radiofrequency ablation	3 (5.2%, *n =* 3/58)
Surgery + Transarterial chemoembolization	1 (1.7%, *n =* 1/58)
Radiofrequency ablation + Transarterial chemoembolization	14 (24.1%, *n =* 14/58)
Transarterial chemoembolization + Radiation therapy	10 (17.2%, *n =* 10/58)
Surgery + Radiofrequency ablation + Transarterial chemoembolization	6 (10.3%, *n =* 6/58)
Radiofrequency ablation + Transarterial chemoembolization + Radiation therapy	6 (10.3%, *n =* 6/58)
Surgery + Transarterial chemoembolization + Radiation therapy	1 (1.7%, *n =* 1/58)
Previous diagnosis of HCC	
Pathologic diagnosis	16 (27.6%, *n =* 16/58)
Image-based diagnosis	42 (72.4%, *n =* 42/58)

HCC, hepatocellular carcinoma; SD, standard deviation.

We also evaluated the clinical data from electronic medical records, such as age, sex, underlying liver disease, history of HCC treatment, tumor marker level (AFP and/or PIVKA-Ⅱ), and results of gadoxetic acid-enhanced MRI and other imaging studies. After treatment, the patients who had achieved a complete response were recommended to continue followed up with liver imaging studies (CT or MRI) and tumor marker measurement every 3 months. When elevated tumor marker levels were encountered in spite of a negative liver MRI, the patients were advised to either 1) immediately add chest CT and whole body bone scan (or alternatively PET-CT) or 2) go for a short term follow up with only liver imaging (CT or MRI) and tumor markers, and then perform chest CT and whole body bone scan (or alternatively perform PET-CT) if the tumor marker levels did not normalize while liver imaging study remained negative. Additional studies such as brain MRI or spine MRI were provided if specific symptoms developed.

Clinical outcomes and results of follow-up imaging studies were obtained through January 2020 to ensure a follow-up interval of at least 15 months for patients enrolled at the latter part of the study.

This was an observational study and statistical analysis was not performed.

### MRI acquisition

MRI was performed using a 3.0T MR unit (Achieva or Ingenia; Philips Healthcare, Best, Netherlands) (Magnetom Skyr; Siemens Healthcare, Erlangen, Germany). Baseline MRI included a T1-weighted turbo field echo in- and opposed-phase sequence, breath-hold multi-shot T2-weighted imaging (T2WI), and respiratory-triggered heavily T2WI. Diffusion-weighted imaging (DWI) was performed using respiratory-triggered, single-shot, echo-planar imaging with b-values of 0, 100, and 800 s/mm^2^. For contrast-enhanced liver dynamic MRI, gadoxetic acid (Primovist; Bayer Healthcare, Berlin, Germany) was administered intravenously using a power injector at a rate of 1 mL/s for a dose of 0.025 mmol/kg body weight, followed by a 20-mL saline flush. Images were obtained precontrast and after injection of the contrast agent in the arterial phase (25–30 seconds), portal phase (60 seconds), transitional phase (3 minutes), and hepatobiliary phase (20 minutes). Detailed parameters of each sequence are summarized in Table 2.

**Table 2:** Representative parameters used for liver MRI.

Sequence	Echo time/ repetition time	Flip angle	Slice thickness (mm)	Matrix Size	Bandwidth (Hz/pixel)	Field of view (cm)	Acquisition time (s)	No. of excitations
T1W-3D dual GRE	3.5/1.15–2.3	10	3	256 × 194	434.4	32–38	14	1
BH-MS-T2WI	1623/70	90	5	324 × 235	235.2	32–38	33/13.7	1
RT-SS-HT2WI	1156/70	90	5	320 × 256	317.9	32–38	120	2
Diffusion-weighted image	1600/70	90	5	112 × 108	79.5	34	126	4
T1W-3D-GRE	3.1/1.5	10	3	256 × 256	995.7	32–38	16.6	1

GRE, gradient echo; BH-MS-T2WI, breath-hold, multi-shot T2-weighted imaging; MRI, magnetic resonance imaging; RT-SS-HT2WI, respiration-triggered single-shot heavily T2-weighted imaging.

## Results

The inclusion and exclusion criteria defined a final study population of 58 patients (mean±standard deviation [SD]: 61.0±10.0 years, range: 27−84 years), including 46 males (mean±SD: 61.0±9.4 years; range: 41−84 years) and 12 females (mean±SD: 60.8±12.1 years; range: 27−76 years) (Table 1). The initial HCC diagnosis was based on pathological analysis in 16 patients and imaging analysis in 42 patients.

Overall, seven false-negative MRI interpretations missed an HCC recurrence. These were initially reported as negative in the official report but contained a recurrent lesion at the second review by the multidisciplinary tumor board. The recurrences included patients with intra-hepatic recurrent HCC (*n =* 2), peritoneal seeding lesions (*n =* 4), and adrenal gland metastasis (*n =* 1). The patients with these detection failures were not included in the final study population (Fig 1).

Our study population consisted of 58 patients with clinically suspected hidden HCC recurrence due to elevated tumor marker levels, but gadoxetic acid-enhanced MRI failed to identify an attributable tumor lesion. All patients had a previous HCC diagnosis but were achieved a complete response after treatment. Additional imaging studies for systemic evaluation, such as positron emission tomography (PET)-CT (*n =* 8), chest CT (*n =* 28), whole-body bone scan (*n =* 28), spine MRI (*n =* 1), and brain MRI (*n =* 1) were also negative.

The tumor markers measured at the time of referral were AFP (median: 15.3; interquartile range: 19.4; range: 1.3–831.4; reference range: 8.1 ng/mL) and PIVKA-Ⅱ (median: 30.5; interquartile range: 72.5; range: 9–1353; reference range: 40 mAU/mL). At pre-treatment period, 96.6% (n = 56/58) of the patients showed increased levels of either AFP (median: 38.8; interquartile range: 186.8; range: 96.2–8617.5) or PIVKA-Ⅱ (median: 67.0; interquartile range: 309.3; range: 11–75000). There was a general tendency of tumor marker levels to decrease after complete response has been achieved (Table 3), and during follow up the tumor markers usually continued to decrease to eventually reached normal range in the majority of patients (82.8%, *n =* 48/58).

**Table 3:** Tumor marker levels (mean ± standard deviation) of each patient subgroup.

	Tumor marker	Early (≤3 months) recurrence		Recurrence beyond 3 months		No recurrence
		(*n =* 24)		(*n =* 28)		(*n =* 6)
		Intra-hepatic recurrence	Extra-hepatic metastasis	Intra-hepatic recurrence	Extra-hepatic metastasis	
Post-treatment levels (at the time of referral)	AFP (ng/mL)	Median: 21.4, IQR: 34.8 (2.5–489.5)	Median: 2.5 (1.3, 2.5, 147)	Median: 13.1, IQR: 11.1 (1.3–82.5)	Median: 28.0, IQR: 26.3 (6.2–831.4)	Median: 9.3, (IQR: 7.4 (2.8–182.8)
	PIVKA-II (mAU/mL)	Median: 42, IQR: 47 (9–280)	Median: 90 (59, 90, 1076)	Median: 29, IQR: 32.5 (9–141)	Median: 18, IQR: 50.5 (12–399)	Median: 38.5, IQR: 79.5 (11–1353)
Post-treatment levels (when the recurrent tumor was detected)	AFP (ng/mL)	Median: 51.3, IQR: 261 (2.2–1278.3)	Median: 2.2 (1.3, 2.2, 149)	Median: 26.8, IQR: 146.3 (1.3–2833.6)	Median: 621.8, IQR: 1569.1 (9.6–2833.6)	
	PIVKA-II (mAU/mL)	Median: 73.5, IQR: 130.3 (11–14199)	Median: 162 (154, 162, 799)	Median: 61, IQR: 271 (15–3499)	Median: 28, IQR: 1874 (19–3528)	
Pre-treatment levels	AFP (ng/mL)	Median: 42.2, IQR: 231.8 (1.9–8617.5)	Median: 71.4 (4.7, 71.4, 75.6)	Median: 37.6, IQR: 181.9 (1.9–5497)	Median: 98.7, IQR: 242.5 (11.5–46399)	Median: 12.3, IQR: 30.2 (3.4–194.4)
	PIVKA-II (mAU/mL):	Median: 87.5, IQR: 248.3 (11–1200)	Median: 137 (50, 137, 75000)	Median: 56, IQR: 399 (14–40325)	Median: 138, IQR: 1966.0 (23–40325)	Median: 28.5, IQR: 88.3 (10–1200)
Post-treatment levels (at the time of complete response)	AFP (ng/mL)	Median: 5.8, IQR: 7.5 (1.3–86.2)	Median: 3.8 (3.1, 3.8, 35.6)	Median: 8.5, IQR: 9.1 (1.9–69.9)	Median: 10.3, IQR: 18.7 (2.1–71.3)	Median: 5.4, IQR: 5.2 (2–25.9)
	PIVKA-II (mAU/mL)	Median: 22.5, IQR: 19.3 (9–418)	Median: 35 (18, 35, 107)	Median: 24, IQR: 11.3 (10–117)	Median: 17, IQR: 3.8 (12–27)	Median: 18.5, IQR: 11.5 (9–89)

AFP, alpha-fetoprotein; PIVKA-II, protein induced by vitamin K absence or antagonist-Ⅱ; IQR, interquartile range.

**Fig 2:**
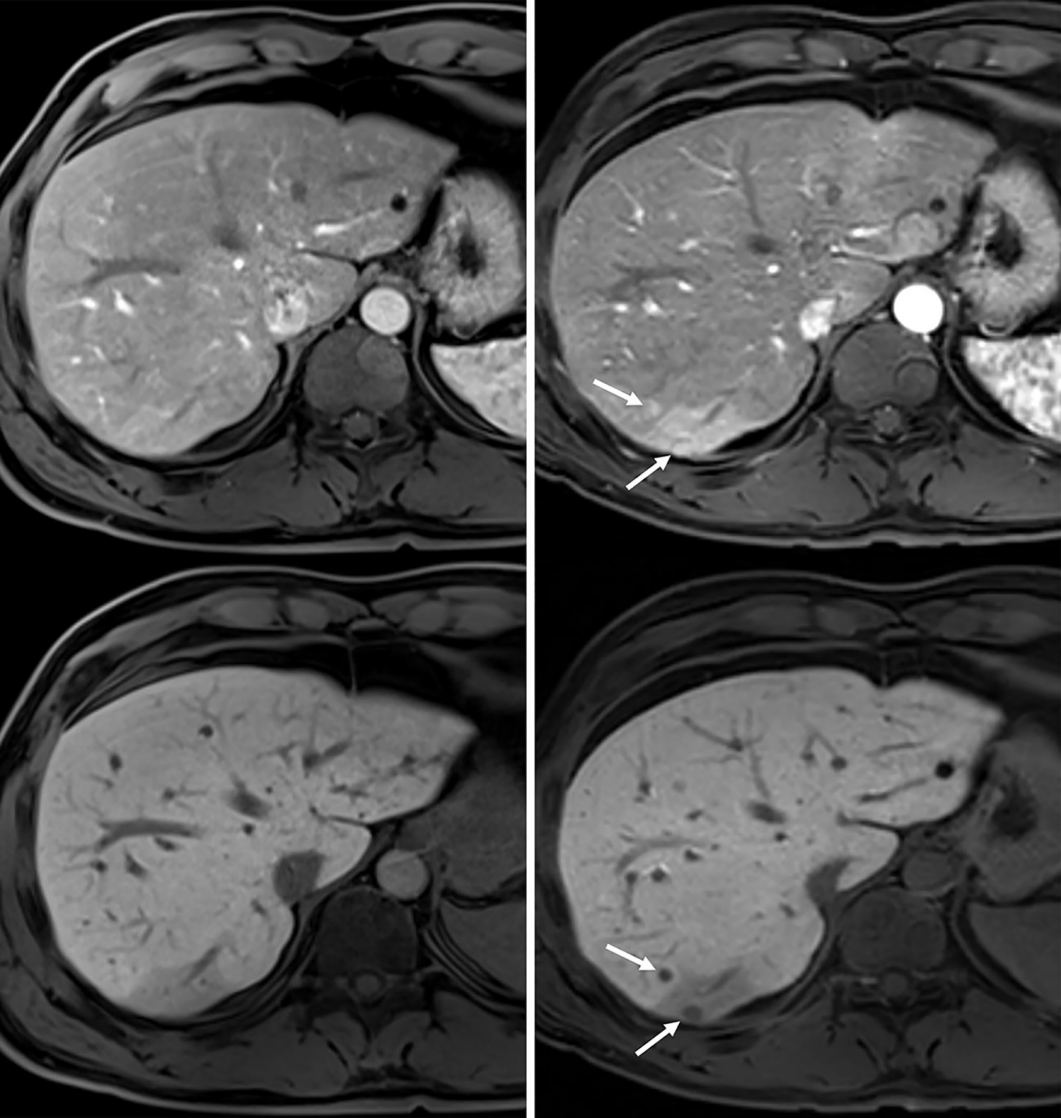
**Gadoxetic acid-enhanced MRI arterial phase (upper) and hepatobiliary phase (lower) images of a 41-year-old man who had undergone liver S6 segmentectomy for hepatocellular carcinoma (HCC).** (**a**) MRI performed 7.6 months after surgery revealed no evidence of HCC recurrence (AFP: 15.1 ng/ml; PIVKA-II: 15 mAU/ml). (**b**) MRI obtained after an additional 48 days revealed two new subcentimeter-sized nodules (arrows) showing arterial phase enhancement and decreased hepatobiliary phase signal intensity, indicating HCC recurrence (AFP: 119.5 ng/ml; PIVKA-II: 26 mAU/ml).

The first follow-up assessment session was usually performed within approximately 3 months after selection by the multidisciplinary tumor board. In 24 patients (41.4%, *n =* 24/58), recurrent HCC was detected during the first short-term follow-up session. In 22 of these patients, an intra-hepatic recurrent lesion was detected (1 patient also had bone metastasis) (Fig 2). These lesions were detected by liver MRI, liver CT, or conventional angiography in 15, 5, and 2 patients, respectively. In the patient who presented with intra-hepatic recurrence and bone metastasis, bone metastasis was detected by PET-CT. In this early intra-hepatic recurrence group, the mean time between initial negative gadoxetic acid-enhanced MRI and the detection of recurrent HCC was 74 days (74±24; range 24–106 days). The median levels of initially elevated AFP and PIVKA-II in these patients were 21.4 ng/mL (interquartile range: 34.8; range: 2.5–489.5) and 42 mAU/mL (interquartile range: 47; range: 9–280), respectively.

Extra-hepatic metastasis within 3 months was found in 3 patients, presenting as lung metastasis alone (*n =* 1), lung and bone metastasis (*n =* 1), or bone metastasis and intra-hepatic recurrence (*n =* 1). All three patients had underwent chest CT and whole body bone scan imaging studies which were found negative for metastasis, either before (during routine follow up after complete response was achieved) or after (as means of systemic evaluation) elevated tumor marker elevation had been detected.

Extra-hepatic metastases were detected by chest CT, spinal MRI, or PET-CT. PET-CT was performed in two patients and successfully detected multiple tumor lesions in the lung, bone as well as intra-hepatic tumor recurrence. The interval between initial gadoxetic acid-enhanced MRI and the detection of distant metastasis were 23, 58, and 73 days in each patient. The initial tumor marker levels were AFP (1.3, 2.5, and 147 ng/mL) and PIVKA-II (59, 90, and 1076 mAU/mL) (Table 3).

**Fig 3:**
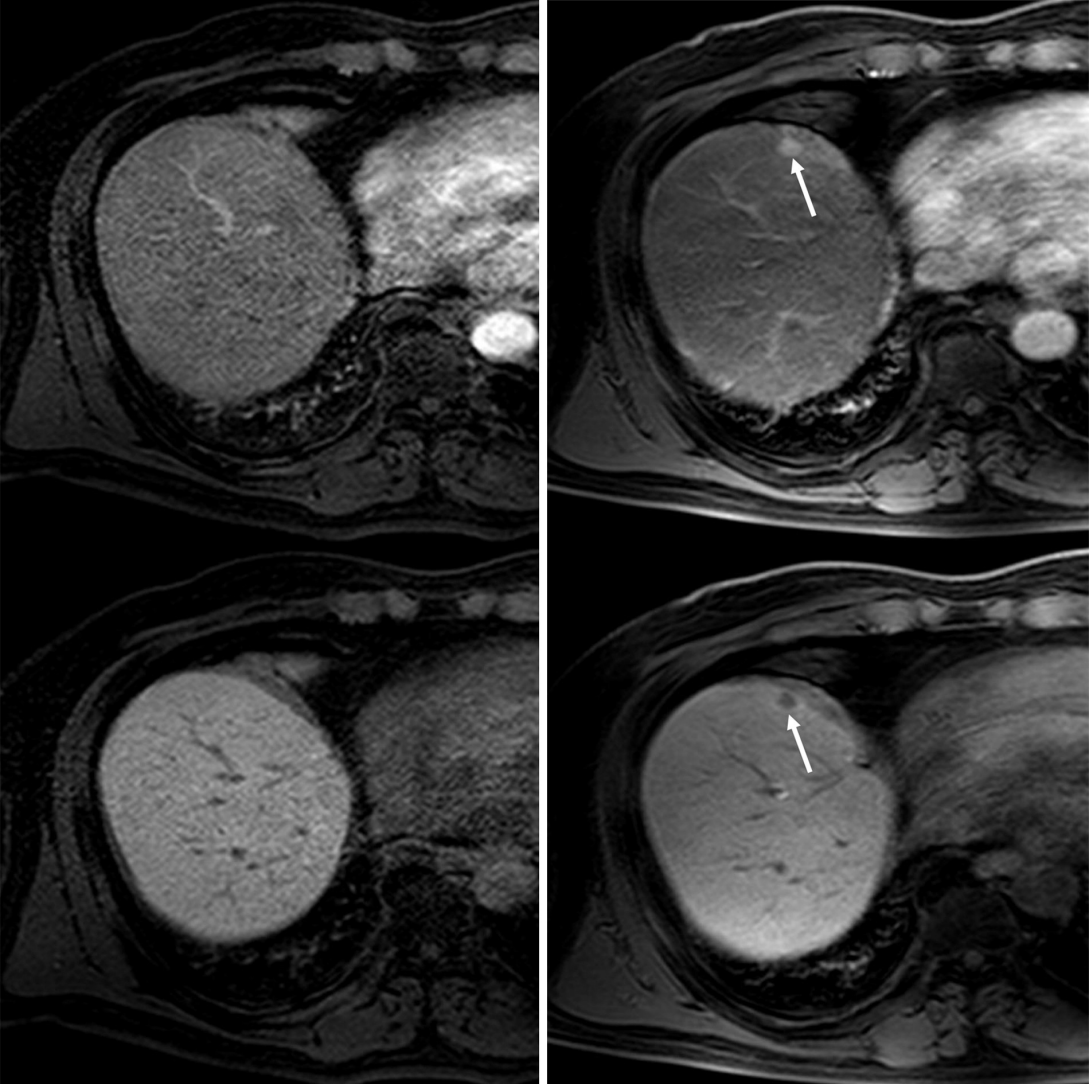
Gadoxetic acid-enhanced MRI arterial phase (upper) and hepatobiliary phase (lower) images of a 60-year-old man who had undergone liver S4 segmentectomy for HCC. **(a)** MRI performed 11 years after surgery revealed no evidence of HCC recurrence (AFP: 2.9 ng/ml; PIVKA-II: 346 mAU/ml). **(b)** After an additional 234 days, MRI demonstrated a newly developed nodule at S8 with arterial phase enhancement (arrow) and low signal intensity on hepatobiliary phase (arrow), indicating HCC recurrence.

After the abovementioned first, short-term follow-up session, 34 patients showed no recurrent HCC on imaging studies. During further follow-up, 23 patients (39.7%, *n =* 23/58) developed recurrent HCC in the liver (2 patients presented with bone metastasis as well) after 3 months (Fig 3). These lesions were detected by either gadoxetic acid-enhanced liver MRI (*n =* 15), liver CT (*n =* 5), and/or conventional angiography (*n =* 3). In this group, the mean period between initial gadoxetic acid-enhanced MRI and the diagnosis of recurrent HCC was 343 days (343±243, range: 130–1219 days). The median levels of initially elevated AFP and PIVKA-II in these patients were 13.1 ng/mL (interquartile range: 11.1; range: 1.3–82.5) and 29 mAU/mL (interquartile range: 32.5; range: 9–141), respectively (Table 3).

Extra-hepatic metastasis after 3 months occurred in 7 patients (12.1%; *n =* 7/58), presenting as bone metastasis and intra-hepatic HCC recurrence together (*n =* 2), bone metastasis alone (*n =* 1), lymph node (*n =* 2), lung (*n =* 1), or brain (*n =* 1) metastasis. These patients underwent chest CT (n = 7), whole body bone scan (n = 7) and PET-CT (n = 2) of which results were negative for metastasis after they had been referred to our multidisciplinary tumor board.

**Fig 4:**
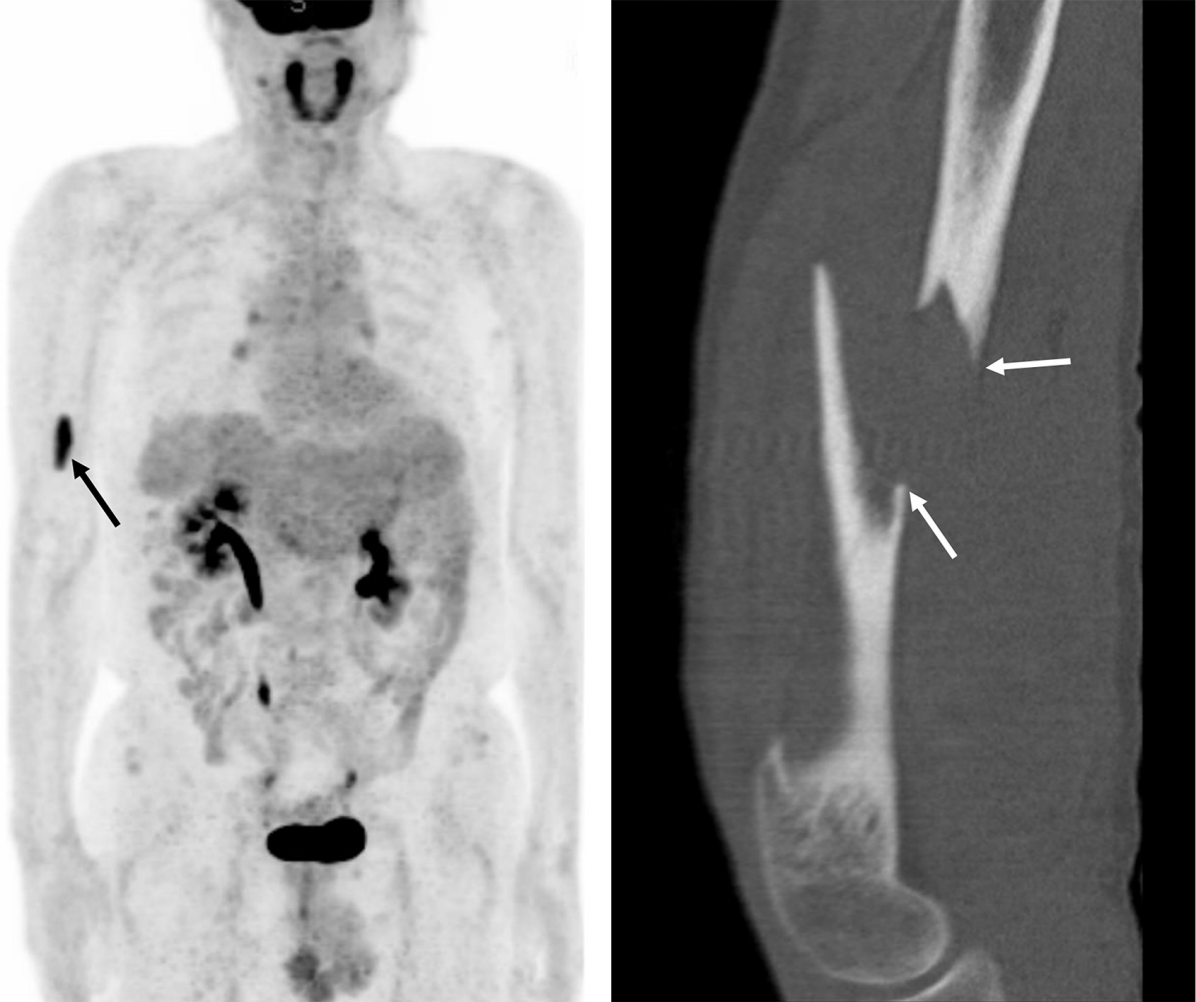
A 76-year-old man who had undergone radiofrequency ablation, transarterial chemoembolization, and proton beam radiation therapy. After 7 months, the tumor marker levels were remeasured (AFP: 2.6 ng/ml; PIVKA-II: 123 mAU/ml), while MRI did not reveal a recurrent lesion. A thorough evaluation was performed including 2 liver MRI scans within 105 days and CT of the abdomen and chest, but all were negative. (**a**) PET-CT (3-dimensional maximum intensity projection imaging) performed 107 days after elevated tumor marker levels were first observed, showing metastasis (arrow) at the right humerus. (**b**) CT scan of the right humerus obtained 5 days after PET-CT when a non-traumatic pathologic fracture (white arrow) occurred during regular physical activity.

Bone metastasis was detected by liver CT with pelvic extension and/or PET-CT (Fig 4). All lymph node metastasis occurred at the hepatoduodenal ligament and was first detected by liver MRI. Lung and brain metastases were first detected by chest CT and brain MRI, respectively. The median follow-up period between the initial MRI to detection of distant metastasis was 236 days (interquartile range: 93; range: 116–658 days). The median levels of initially elevated AFP and PIVKA-II in these patients were 28.0 ng/mL (interquartile range: 26.3; range: 6.2–831.4) and 18 mAU/mL (interquartile range: 50.5; range: 12–399), respectively (Table 3).

In the six remaining patients (10.3%; *n =* 6/58), a recurrent tumor was not detected for at least 15 months during additional follow-up. The median follow-up period of these patients was 596 days (interquartile range: 49.5; range: 554–2206 days). The median levels of initially elevated AFP and PIVKA-II in these patients with no recurrence were 9.3 ng/mL (interquartile range: 7.4; range: 2.8–182.8) and 38.5 mAU/mL (interquartile range:79.5; range: 11–1353), respectively (Table 3).

## Discussion

Kim et al. reported the outcome of high-risk patients not previously diagnosed with HCC who presented with elevated AFP levels but negative findings on MRI during screening [16]. In their study, 17 high-risk patients without a history of HCC and presenting with AFP levels greater than 300 ng/mL but negative gadobenate dimeglumine- or gadodiamide-enhanced MRI were followed, and 59% (*n =* 10/17) developed HCC in the liver after a mean of 138 days (range: 41–247 days) [16].

In a similar but different context, we examined patients with HCC who had achieved a complete response to treatment and had undergone gadoxetic acid-enhanced MRI. In our study recurrent HCC was initially suspected based on a surge of tumor markers which had been suppressed during follow up period. Although there are some similarities to screening high-risk patients without previous HCC, we believe that imaging surveillance of patients treated for HCC should differ in a few aspects. In high-risk patients without previous HCC, imaging studies limited to the liver will usually be sufficient to screen for primary HCC. However, HCC recurrence can present as 1) recurrence at a site of incomplete initial treatment, 2) micrometastasis outside the treated field, or 3) new cancer in the form of a second primary lesion [20]. As shown in our results, metastasis can be both intra-hepatic and extra-hepatic. Therefore, patients with treated HCC should be considered high risk even with a complete response, and appropriate surveillance for both types of recurrence is necessary.

**Fig 5:**
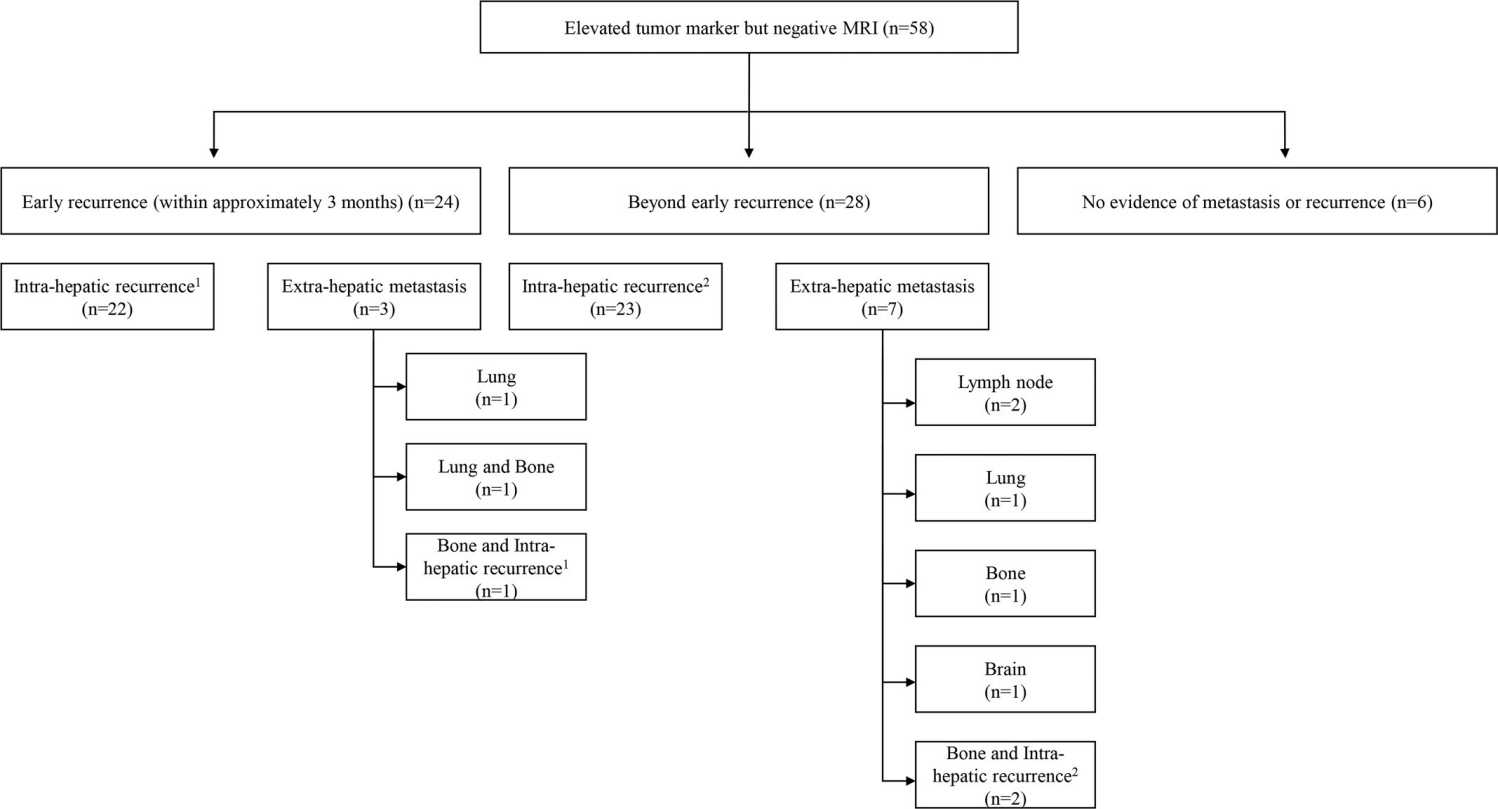
Schematic summary of the clinical outcomes of the study population. Some patients presented with both intra-hepatic recurrence and bone metastasis in the early recurrence (*n =* 1)^1^ and beyond early recurrence (*n =* 2)^2^ groups.

Our study population was mainly comprised of HCC patients who had achieved a complete response to treatment but later presented with elevated tumor marker levels without an identifiable recurrence on gadoxetic acid-enhanced MRI. Of these patients, 89.7% (*n =* 52/58) developed HCC within the study period. Notably, approximately half of the patients who developed recurrent tumors (46.2%; *n =* 24/52) presented with a detectable lesion within 3 months (Fig 5). The most frequent site of recurrence was the liver (86.5%; *n =* 45/52; including 3 patients with both intra-hepatic recurrence and extra-hepatic metastases), but extra-hepatic metastasis was also common (19.2%; *n =* 10/52; including 3 patients with both intra-hepatic recurrence and extra-hepatic metastasis) (Fig 5). In 5.8% (*n =* 3/52) of these patients, the newly detected HCC occurred as combined intra-hepatic recurrence and extra-hepatic metastasis. Based on these observations, we suggest that liver MRI should be repeated within 3 months along with liver CT with pelvic extension and chest CT. If these studies remain negative, we recommend additional short-term follow-up with imaging studies, although the duration of this approach is unknown. However, a previous study reported that an elevated level of preoperative AFP and/or PIVKA-II correlated with early postoperative recurrence (≤6 months) because early phase recurrence represents metastasis rather than a secondary de novo tumor [13]. Based on this information, we believe that such intense imaging follow-up strategies should be maintained for no less than 6 months.

Another consideration during surveillance is that typical HCC imaging can become deranged after treatment, possibly causing MRI misinterpretation. Reactive hyperemia can occur around the treated area of radiofrequency ablation and sometimes may be confused with tumor enhancement [21]. After stereotactic body radiation therapy, arterial phase hyperenhancement may persist but does not necessarily indicate the presence of residual viable HCC [22, 23]. Additionally, peritumoral liver arterial phase enhancement or delayed enhancement commonly occurs likely because of sinusoidal congestion and liver parenchymal inflammation followed by fibrosis [19, 23]. We believe that these lesions can obscure small recurrent HCCs and theoretically explain false-negative MRI results. Although considerable progress has been achieved in the MRI assessment of HCC recurrence after treatment [24–26], further studies on image interpretation after treatment and recurrent HCC are necessary.

Our study has a few limitations. First, this study was retrospective in nature, with the study population extracted from a pool of patients referred to our multidisciplinary tumor board by physicians. We recognize that this process might have recruited patients with more challenging situations, thereby increasing selection bias. Second, our study population was heterogeneous in terms of the baseline HCC stage and previous treatment type. Third, in most patients, a pathological diagnosis was not achieved for the initial HCC diagnosis. Instead, the diagnosis of patients was established based on typical imaging findings and the clinical course. Fourth, we only performed gadoxetic acid-enhanced MRI. Therefore, our conclusions may not be directly translatable at centers that use extracellular contrast agents for liver MRI.

In conclusion, an increased tumor marker level encountered during the surveillance of patients with a complete response to HCC treatment indicates a high probability of impending tumor recurrence even if gadoxetic acid-enhanced MRI fails to detect a recurrent tumor. We suggest short-term follow-up with liver MRI within 3 months, as well as a systemic evaluation by chest and liver CT with pelvic extension; if these studies are negative, they should be repeated after additional short-term follow up.

## Abbreviations

AFP: Alpha-fetoprotein
HCC: Hepatocellular carcinoma
PIVKA-Ⅱ: protein induced by vitamin K absence or antagonist-Ⅱ
